# New Delhi Metallo-Beta-Lactamase-Producing Enterobacteriaceae in South Korea Between 2010 and 2015

**DOI:** 10.3389/fmicb.2018.00571

**Published:** 2018-03-29

**Authors:** Eun-Jeong Yoon, Da Young Kang, Ji Woo Yang, Dokyun Kim, Hyukmin Lee, Kwang Jun Lee, Seok Hoon Jeong

**Affiliations:** ^1^College of Medicine, Yonsei University, Seoul, South Korea; ^2^College of Health Science, Sangji University, Wonju, South Korea; ^3^Korea Centers for Disease Control and Prevention, Korea National Institute of Health, Cheongju, South Korea

**Keywords:** New Delhi metallo-beta-lactamase, carbapenemase-producing Enterobacteriaceae, IncX3, IncFII, Tn*125*

## Abstract

This study was carried out to investigate the epidemiological time-course of New Delhi metallo-beta-lactamase- (NDM-) mediated carbapenem resistance in Enterobacteriaceae in South Korea. A total of 146 non-duplicate NDM-producing Enterobacteriaceae recovered between 2010 and 2015 were voluntarily collected from 33 general hospitals and confirmed by PCR. The species were identified by sequences of the 16S rDNA. Antimicrobial susceptibility was determined either by the disk diffusion method or by broth microdilution, and the carbapenem MICs were determined by agar dilution. Then, multilocus sequence typing and PCR-based replicon typing was carried out. Co-carried genes for drug resistance were identified by PCR and sequencing. The entire genomes of eight random selected NDM producers were sequenced. A total of 69 *Klebsiella pneumoniae* of 12 sequence types (STs), 34 *Escherichia coli* of 15 STs, 28 *Enterobacter* spp. (including one *Enterobacter aerogenes*), nine *Citrobacter freundii*, four *Raoultella* spp., and two *Klebsiella oxytoca* isolates produced either NDM-1 (*n* = 126), NDM-5 (*n* = 18), or NDM-7 (*n* = 2). The isolates co-produced CTX-M-type ESBL (52.1%), AmpCs (27.4%), additional carbapenemases (7.1%), and/or 16S rRNA methyltransferases (4.8%), resulting in multidrug-resistance (47.9%) or extensively drug-resistance (52.1%). Among plasmids harboring *bla*_NDM_, IncX3 was predominant (77.4%), followed by the IncFII type (5.8%). Genome analysis revealed inter-species and inter-strain horizontal gene transfer of the plasmid. Both clonal dissemination and plasmid transfer contributed to the wide dissemination of NDM producers in South Korea.

## Introduction

The first New Delhi metallo-beta-lactamase (MBL), NDM-1, was identified in 2008 in an extensively drug-resistant (XDR) *Klebsiella pneumoniae* clinical isolate recovered from a urinary specimen of a patient (Yong et al., 2009). The enzyme had an astonishing range of beta-lactam substrates, including penicillins, late-generation cephalosporins, and carbapenems with the exception of monobactams (Yong et al., 2009). The coding gene was found to be located in a ~180-kb plasmid that is readily transferable by conjugation (Yong et al., 2009). Further epidemiology revealed that the gene had already spread to various species of bacteria including *K. pneumoniae, Escherichia coli, Enterobacter* spp., *Morganella morganii*, and *Acinetobacter baumannii* throughout the six continents (Johnson and Woodford, 2013), implying that the plasmid encoding NDM has an enormous ability to spread.

Since the identification of the first NDM variant (NDM-2) in *A. baumannii* (Kaase et al., 2011), 17 subtypes of NDM variants have been functionally characterized (Liu et al., 2017). The NDM subtypes, which differ from the NDM-1 prototype by one to five amino-acid substitutions, exhibit different levels of hydrolyzing activity against carbapenems and other beta-lactam substrates, depending on the substitutions. Among the first seven subtypes, NDM-7 containing two substitutions of Asp130Asn and Met154Leu has the greatest carbapenem-hydrolyzing activity, followed by NDM-5 with Val88Leu and Met154Leu, NDM-6 with Ala233Val, and NDM-1 (Rahman et al., 2014).

The *bla*_NDM_ gene has been found in plasmids of diverse replicon types, such as IncF, IncX3, IncL/M, IncH, and IncA/C, and has an extensive host range. In plasmids, the gene is frequently associated with mobile genetic elements such as transposons and insertion sequences (ISs). The complete form of the transposable element of the *bla*_NDM_ gene Tn*125* was first identified in *A. baumannii*, suggesting that *Acinetobacter* spp. are the reservoir of the gene (Poirel et al., 2012). Tn*125* is bracketed by two copies of IS*Aba125*. One copy of IS*Aba125* is located upstream of the *bla*_NDM_ gene, and the *ble*_MBL_ gene encoding resistance to the anti-tumor glycopeptide bleomycin is at 3′ end of the gene, followed by eight ORFs and the 3′ copy of IS*Aba125*. The composite form of the transposon is common in *A. baumannii*, and at least in this species, the mobility of the *bla*_NDM_ gene is mainly attributed to the transposon (Poirel et al., 2012). Unlike in *A. baumannii*, the Tn*125* transposon of Enterobacteriaceae is in a truncated form that is no longer mobile. This indicates that plasmids are important for *bla*_NDM_ mobility among Enterobacteriaceae (Partridge and Iredell, 2012).

To monitor the spread of carbapenemase-producing Enterobacteriaceae (CPE), the Korea Center for Disease Prevention and Control (KCDC) had run a national surveillance system since 2010. In this study, the NDM producers collected from this surveillance system between 2010 and 2015 were investigated for the epidemiological time-course of NDM-mediated carbapenem resistance by analyzing the clones, plasmids, and genetic contexts of the *bla*_NDM_ gene. In order to evaluate the inter-species and inter-strain horizontal gene transfer, WGS was performed to characterize the main mobile genetic elements responsible for NDM dissemination.

## Materials and methods

### Bacterial strains

During the period between 2010 and 2015, 146 non-duplicate NDM producers were voluntarily collected from 33 general hospitals composing the National Laboratory Surveillance System for carbapenemase-producing Enterobacteriaceae (CPE) of the Korea Center for Disease Prevention and Control. Further testing including the species identification by using Bruker MALDI MS instrument (Bruker, Billerica, MA, USA) and by 16S rDNA sequencing was performed in a reference laboratory.

### Antimicrobial susceptibility testing and resistance genotyping

Antimicrobial susceptibility to eight drugs (aztreonam, cefotaxime, ceftazidime, cefoxitin, gentamicin, amikacin, ciprofloxacin, and tigecycline) was evaluated by the disk diffusion method on Mueller-Hinton (MH) agar (Difco Laboratories, Detroit, MI, USA) in accordance with CLSI guidelines (CLSI, 2015b). The MICs for colistin were assessed by the broth microdilution method with MH broth (Difco Laboratories) in accordance with the recommendations of The joint CLSI–EUCAST (2016). The MICs for imipenem and meropenem were determined by the agar dilution method in accordance with CLSI guidelines (CLSI, 2015a). Both *E. coli* ATCC 25922 and *Pseudomonas aeruginosa* ATCC 27853 were used for quality control. The *bla*_NDM_ gene was detected by PCR with gene-specific primers and direct sequencing was carried out for NDM subtyping (Poirel et al., 2011). Genetic contexts for the vicinity of the *bla*_NDM_ gene were explored by PCR mapping using primers in the supplemental figure. The confirmed NDM producers were further evaluated by PCR and by direct sequencing to identify the following additional resistance determinants: *bla*_OXA−48−like_, *bla*_IMP_, *bla*_VIM_, and *bla*_GES_ for carbapenemases (Poirel et al., 2011); *bla*_CTX−M−1−like_, *bla*_CTX−M−9−like_, and *bla*_CTX−M−25−like_ for CTX-M-type extended-spectrum-beta-lactamases (ESBLs); *bla*_ACT_, *bla*_ACC_, *bla*_CMY_, and *bla*_DHA_ for plasmid-mediated AmpCs; and *armA, rmtA*, and *rmtB* for 16S ribosomal methyltransferases (Pérez-Pérez and Hanson, 2002; Ryoo et al., 2005).

### MLST

PCR and sequencing were carried out for seven housekeeping genes per species: *gapA, infB, mdh, pgi, phoE, rpoB*, and *tonB* for *K. pneumoniae* (Diancourt et al., 2005) and *adk, fumC, gyrB, icd, mdh, purA*, and *recA* for *E. coli* (Wirth et al., 2006) and the sequences were compared in the multilocus sequence typing (MLST) database, so that allelic numbers and sequence types (STs) could be determined.

### Plasmid transfer by bacterial conjugation and electroporation

For bacterial conjugation, spontaneous rifampin-resistant mutants from *E. coli* J53 were used as recipients. Equal amounts of exponential cultures of the donor and recipient isolates were mixed, incubated in MH broth for 12 h, and spread on Brain Heart Infusion (BHI) agar (Difco Laboratories) containing rifampin (30 mg/L), sodium azide (100 mg/L), and imipenem (1 mg/L). Each colony was tested by disk diffusion test and confirmed by PCR. The plasmid transfer frequency was calculated as the number of transconjugants per donor. The non-conjugative plasmids were electrotransferred into One Shot^TM^ TOP10 Electrocomp^TM^ *E. coli* (ThermoFisher Scientific, Waltham, MA, USA) following the manufacturer's instruction and selected on BHI supplemented with imipenem 4 mg/L.

### Whole genome sequencing

Eight NDM producers were entirely sequenced: one *Enterobacter* sp. with an IncX3 plasmid encoding NDM-1; four *E. coli* isolates with IncX3 plasmids encoding any of NDM-1, −5, and −7; one *Citrobacter freundii* with IncX3 plasmid encoding NDM-5; two *K. pneumoniae* strains co-producing OXA-181 and NDM-5 which the *bla*_NDM−5_ gene was carried by the IncFII plasmid. Bacterial whole genomes were sequenced with single-molecule real-time sequencing on a PacBio RSII instrument (Pacific Biosciences, Menlo Park, CA, USA) as previously described (Hong et al., 2016). *De novo* genome assembly was performed with the PacBio SMRT analysis software suite (version 2.3.0) and the annotations of coding sequences, tRNA sequences, and rRNA sequences were performed using the NCBI Prokaryotic Genome Annotation Pipeline.

### GenBank accession

The genomes of eight Enterobacteriaceae isolates are available in the GenBank nucleotide database under the accession numbers CP024812-CP024814 for *Enterobacter* sp. CRENT-193, CP024815-CP024818 for *E. coli* CREC-629, CP024819- CP024820 for *C. freundii* for CRCB-101, CP024821-CP024825 for *E. coli* CREC-591, CP024826-CP024829 for *E. coli* CREC-544, CP024830-CP024833 for *E. coli* CREC-532, CP024834-CP024837 for *K. pneumoniae* CRKP-2297, and CP024838-CP024841 for *K. pneumoniae* CRKP-1215 under the BioProject PRJNA416193.

## Results

### Occurrence of NDM producers between 2010 and 2015

In 2010, four NDM-1-producing *K. pneumoniae* ST340 isolates were identified in a single hospital outbreak in Seoul (Kim et al., 2012). In 2011, five isolates of the same clone were successively identified at the same hospital and one and three NDM-1-producing *E. coli* ST101 isolates were identified at another hospital in Seoul and at a hospital in the southeastern region of the Korean peninsula, respectively. The NDM-1-producing *K. pneumoniae* ST340 was identified in the central part of the peninsula in 2012 and in 2013, diverse NDM-producing bacterial pathogens such as *C. freundii* and *Enterobacter* spp. were identified in a broader region, including the southern peninsula. By 2014, the number of NDM producers had explosively increased from eight to 22, with even more diverse bacterial pathogens, including *Klebsiella oxytoca* and *Raoultella planticola*. Finally, in 2015, among the 102 NDM producers identified over the peninsula, NDM-5 and NDM-7 producers were identified. Three of the 18 NDM-5 producers were previously reported as NDM-5 and OXA-181 co-producer (Sun et al., 2016).

### Characteristics of the NDM producers

PCR and sequencing revealed that the 146 NDM-producing Enterobacteriaceae isolates contained either *bla*_NDM−1_ (*n* = 126, 86.3%), *bla*_NDM−5_ (*n* = 18, 12.3%), or *bla*_NDM−7_ (*n* = 2, 1.4%). The isolates included 69 *K. pneumoniae* (47.3%), 34 *E. coli* (23.3%), 27 *Enterobacter* spp. (18.5%), two *K. oxytoca* (1.4%), nine *C. freundii* (6.2%), three *R. planticola* (2.1%), and one *Raoultella ornithinolytica* (0.7%), which were recovered from urine (*n* = 57, 39.0%), pulmonary samples (*n* = 29, 19.9%), rectal swabs (*n* = 22, 15.1%), body fluids (*n* = 15, 10.3%), blood (*n* = 7, 4.8%), wounds (*n* = 7, 4.8%), and others (*n* = 9, 6.2%).

MLST analysis revealed 12 *K. pneumoniae* STs for the 69 isolates: ST1061 (*n* = 20, 29.0%), ST340 (*n* = 10, 14.5%), ST147 (*n* = 9, 13.0%), ST14 (*n* = 8, 11.6%), ST307 (*n* = 7, 10.1%), ST11 (*n* = 6, 8.7%), ST2 (*n* = 4, 5.8%), and one each of ST631, ST70, ST789, ST392, and ST2353. It also identified 15 *E. coli* STs for 34 isolates: ST101 (*n* = 15, 44.1%), ST167 (*n* = 3, 8.8%), ST410 (*n* = 3, 8.8%), ST457 (*n* = 2, 5.9%), and one each of ST38, ST69, ST88, ST90, ST118, ST354, ST361, ST362, ST1177, ST1284, and ST1543. It is worth remarking that *K. pneumoniae* ST340, ST14, ST147, and ST11, along with *E. coli* ST69, are prominent high-risk clones known to be resistant to multiple drugs (Mathers et al., 2015). The group of 18 NDM-5 producers consisted of 14 *E. coli* isolates (77.8%, nine ST101 and one each of ST162, ST361, ST362, ST88, and ST90), three *K. pneumoniae* isolates (16.7%, two ST147 and one ST392), and one *C. freundii* isolate (5.6%), while both NDM-7 producers were *E. coli* ST167.

The isolates were all resistant to both carbapenems, having MICs ≥2 mg/L, except for one isolate that had MICs of 0.5 and 1 mg/L for imipenem and meropenem, respectively (Table 1). The imipenem MIC_50_ and MIC_90_ were 16 and 64 mg/L, respectively, and those of meropenem were 16 and 128 mg/L. Based on the standard definitions for acquired resistance (Magiorakos et al., 2012), all studied NDM producers were either multidrug resistant (MDR; 70/146, 47.9%) or XDR (76/146, 52.1%) (Table 2). No isolate was pan-drug-resistant, as they all remained susceptible to tigecycline or colistin. Ciprofloxacin was the most co-resistant antibiotic (122/146, 83.6%), followed by aztreonam (107/146, 73.3%), even though NDM does not hydrolyze monobactams.

**Table 1:** Distribution of carbapenem MICs in NDM producers by subtype.

	**Number (cumulative %) of isolates having MICs (mg/L) of^a^:**									
	**0.5**	**1**	**2**	**4**	**8**	**16**	**32**	**64**	**128**	**>128**
**IMIPENEM**										
All NDM producers (*n* = 146)	1 (0.7)	0 (0.7)	2 (2.1)	13 (11.0)	34 (34.2)	**45 (65.1)**	26 (82.9)	**15 (93.2)**	5 (96.6)	5 (100)
NDM-1 producers (*n* = 126)	1 (0.8)	0 (0.8)	2 (2.4)	13 (12.7)	34 (39.7)	**37 (69.0)**	19 (84.1)	**13 (94.4)**	2 (96.0)	5 (100)
NDM-5 producers (*n* = 18)	0 (0.0)	0 (0.0)	0 (0.0)	0 (0.0)	0 (0.0)	7 (38.9)	**7 (77.8)**	2 (88.9)	**2 (100)**	0 (100)
NDM-7 producers (*n* = 2)	0	0	0	0	0	1	0	0	1	0
**MEROPENEM**										
All NDM producers (*n* = 146)	0 (0.0)	1 (0.7)	3 (2.7)	9 (8.9)	33 (31.5)	**34 (54.8)**	31 (76.0)	13 (84.9)	**16 (95.9)**	6 (100)
NDM-1 producers (*n* = 126)	0 (0.0)	1 (0.8)	3 (3.2)	9 (10.3)	31 (34.9)	**33 (61.1)**	23 (79.4)	11 (88.1)	**11 (96.8)**	4 (100)
NDM-5 producers (*n* = 18)	0 (0.0)	0 (0.0)	0 (0.0)	0 (0.0)	2 (11.1)	1 (16.7)	**7 (55.6)**	2 (66.7)	**5 (94.4)**	1 (100)
NDM-7 producers (*n* = 2)	0	0	0	0	0	0	1	0	0	1

a *MIC_50_ and MIC_90_ are indicated in bold*.

**Table 2:** Antibiograms of NDM producers by subtype.

	**MDR (%)**	**XDR (%)**	**PDR (%)**	**Number (%) of isolates non-susceptible to:**								
				**CIP**	**GM**	**AN**	**ATM**	**CTX**	**CAZ**	**FOX**	**TGC**	**COL**
All NDM producers (*n* = 146)	70 (47.9)	76 (52.1)	0 (0)	122 (83.6)	86 (58.9)	61 (41.8)	107 (73.3)	146 (100)	146 (100)	146 (100)	9 (6.2)	13 (8.9)
NDM-1 producers (*n* = 126)	56 (44.4)	70 (55.6)	0 (0)	104 (82.5)	78 (61.9)	57 (45.2)	91 (72.2)	126 (100)	126 (100)	126 (100)	8 (6.3)	12 (9.5)
NDM-5 producers (*n* = 18)	14 (77.8)	4 (22.2)	0 (0)	16 (88.9)	6 (33.3)	4 (22.2)	14 (77.8)	18 (100)	18 (100)	18 (100)	1 (5.6)	1 (5.6)
NDM-7 producers (*n* = 2)	0 (0.0)	2 (100)	0 (0)	2 (100)	2 (100)	0 (0.0)	2 (100)	2 (100)	2 (100)	2 (100)	0 (0.0)	0 (0.0)

*MDR, Multidrug-resistant; XDR, extensively drug-resistant; PDR, pan-drug-resistant; CIP, ciprofloxacin; GM, gentamicin; AN, amikacin; ATM, aztreonam; CTX, cefotaxime; CAZ, ceftazidime; FOX, cefoxitin; TGC, tigecycline; COL, colistin*.

The high rate of co-resistance was well-explained by co-harbored genes for antimicrobial resistance (Table 3). Among the NDM producers, 62.3% (91/146) and 39.0% (57/146) harbored at least one gene for ESBL or for plasmid-mediated AmpC, respectively. In addition, 7.5% (11/146) carried another carbapenemase gene and 32.2% (47/146) possessed a gene for 16S rRNA methyltransferase. Most of the NDM-5 producers (15/18) co-produced CTX-M group 1 ESBL and both NDM-7 producers co-produced the CTX-M group 9 ESBL.

**Table 3:** Co-harbored antimicrobial resistance genes in NDM producers.

	**NDM-1 (*n* = 126)**	**NDM-5 (*n* = 18)**	**NDM-7 (*n* = 2)**	**Total (*n* = 146)**
**CTX-M-TYPE EXTENDED-SPECTRUM BETA-LACTAMASE**				
CTX-M group 1	53 (42.1)	13 (72.2)	0 (0.0)	66 (45.2)
*bla*_CTX−M−15_	26 (20.6)	13 (72.2)	0 (0.0)	39 (26.7)
*bla*_CTX−M−22_	2 (1.6)	0 (0.0)	0 (0.0)	2 (1.4)
*bla*_CTX−M−28_	19 (15.1)	0 (0.0)	0 (0.0)	19 (13.0)
*bla*_CTX−M−3_	3 (2.4)	0 (0.0)	0 (0.0)	3 (2.1)
*bla*_CTX−M−55_	3 (2.4)	0 (0.0)	0 (0.0)	3 (2.1)
CTX-M group 9	7 (5.6)	1 (5.6)	1 (50.0)	9 (6.2)
*bla*_CTX−M−9_	3 (2.4)	0 (0.0)	0 (0.0)	3 (2.1)
*bla*_CTX−M−14_	3 (2.4)	1 (5.6)	1 (50.0)	5 (3.4)
*bla*_CTX−M−27_	1 (0.8)	0 (0.0)	0 (0.0)	1 (0.7)
Group 1 and group 9	0 (0.0)	0 (0.0)	1 (50.0)	1 (0.7)
*bla*_CTX−M−55_ / *bla*_CTX−M−14_	0 (0.0)	0 (0.0)	1 (50.0)	1 (0.7)
**PLASMID-MEDIATED AmpC**				
ACT type	2 (1.6)	0 (0.0)	0 (0.0)	2 (1.4)
*bla*_ACT−1_	2 (1.6)	0 (0.0)	0 (0.0)	2 (1.4)
CMY type	8 (6.3)	1 (5.6)	0 (0.0)	9 (6.2)
*bla*_CMY−2_	5 (4.0)	1 (5.6)	0 (0.0)	6 (4.1)
*bla*_CMY−42_	1 (0.8)	0 (0.0)	0 (0.0)	1 (0.7)
*bla*_CMY−48_	1 (0.8)	0 (0.0)	0 (0.0)	1 (0.7)
*bla*_CMY−64_	1 (0.8)	0 (0.0)	0 (0.0)	1 (0.7)
DHA type	25 (19.8)	0 (0.0)	0 (0.0)	25 (17.1)
*bla*_DHA−1_	25 (19.8)	0 (0.0)	0 (0.0)	25 (17.1)
CMY type and DHA type	4 (3.2)	0 (0.0)	0 (0.0)	4 (2.7)
*bla*_CMY−4_ / *bla*_DHA−1_	4 (3.2)	0 (0.0)	0 (0.0)	4 (2.7)
**CARBAPENEMASES**				
*bla*_KPC−2_	3 (2.4)	0 (0.0)	0 (0.0)	3 (2.1)
*bla*_KPC−4_	1 (0.8)	0 (0.0)	0 (0.0)	1 (0.7)
*bla*_OXA−181_	0 (0.0)	2 (11.1)	0 (0.0)	2 (2.7)
*bla*_OXA−232_	3 (2.4)	1 (5.6)	0 (0.0)	4 (2.7)
*bla*_VIM−2_	1 (0.8)	0 (0.0)	0 (0.0)	1 (0.7)
**16S rRNA METHYLTRANSFERASE**				
*armA*	41 (32.5)	3 (16.7)	0 (0.0)	44 (30.1)
*rmtC*	2 (1.6)	0 (0.0)	0 (0.0)	2 (1.4)
*rmtF*	0 (0.0)	1 (5.6)	0 (0.0)	1 (0.7)

### The *bla*_NDM_-carrying plasmids

PCR-based replicon typing (Carattoli et al., 2005) after conjugation or electroporation allowed us to scrutinize the bacterial dissemination of plasmids belonging to four different incompatibility types: IncFII (*n* = 9, 5.8%), IncX3 (*n* = 113, 77.4%), IncA/C (*n* = 5, 3.4%), and non-typeable (*n* = 19, 13.0%). Among these plasmids, only IncX3 was carried by diverse bacterial species including *K. pneumoniae, E. coli, Enterobacter* spp., *K. oxytoca*, and *C. freundii*, highlighting its broad host range. The *bla*_NDM−1_ gene was carried by all four types of plasmids, while *bla*_NDM−5_ was carried by either IncFII or IncX3 plasmids, and *bla*_NDM−7_ was carried by the IncX3 plasmid.

By liquid mating, plasmid transfer was detected in only one third (29.5%, 43/146) of the plasmids with various conjugation efficiencies (from 5 × 10^−5^ to 5 × 10^−8^) and the rest (70.5%, 103/146) presented plasmid transfer frequencies under the detection threshold (<1 × 10^−9^). Plasmids belonging to the same incompatibility type resulted in varied rate of plasmid transfer: 28.3% (32/113) of IncX3 plasmids had transfer frequencies of 1 × 10^−6^ to 1 × 10^−8^, while 71.7% (81/113) of those presented below the detection threshold; two of the six (33.3%) IncFII plasmids had transfer rates either of 5 × 10^−5^ or 3 × 10^−7^ and the other four (66.7%) presented below the detection threshold; and one of the five (20%) IncA/C plasmids had plasmid transfer frequency of 1 × 10^−5^ and the other four had undeterminable rate.

Since the genetic environment of the *bla*_NDM_ gene is diverse in terms of size and Tn*125* truncation, the vicinity of the gene was evaluated by PCR mapping. For the upstream region of the gene, all plasmids had a promoter region located at the 72-bp extremity of IS*Aba125*. One exceptional case having imipenem and meropenem MICs of 16 and 32 mg/L, respectively, carried a relatively short IS*125* remnant at the upstream of the *bla*_NDM_ gene, remaining the promoter sequences. Downstream of the gene, 17 cases of truncated Tn*125* ended with the *groEL* gene (the 6th ORF from *bla*_NDM_ among eight) and thus the most preserved, whereas one case ended with *ble*_MBL_ next to the *bla*_NDM_ gene (the most shrunken case). Around half of the cases (*n* = 66, 45.2%) ended with the *tat* gene, the 4th ORF from the *bla*_NDM_ gene, while 34.2% (*n* = 50) terminated with *trpF*, the 2nd ORF from the gene. The Tn*125* transposons carrying *bla*_NDM−1_ were truncated at heterogeneous sites along the transposon, while those carrying *bla*_NDM−7_ ceased at the *tat* gene and those carrying *bla*_NDM−5_ were truncated at the end of either *tat* or *trpF*. No obvious correlation was observed between carbapenem susceptibility and the Tn*125* truncation locus.

### IncX3 plasmids carrying three subtypes of *bla*_NDM_ and IncFII plasmids carrying *bla*_NDM−5_

**Figure 1:**
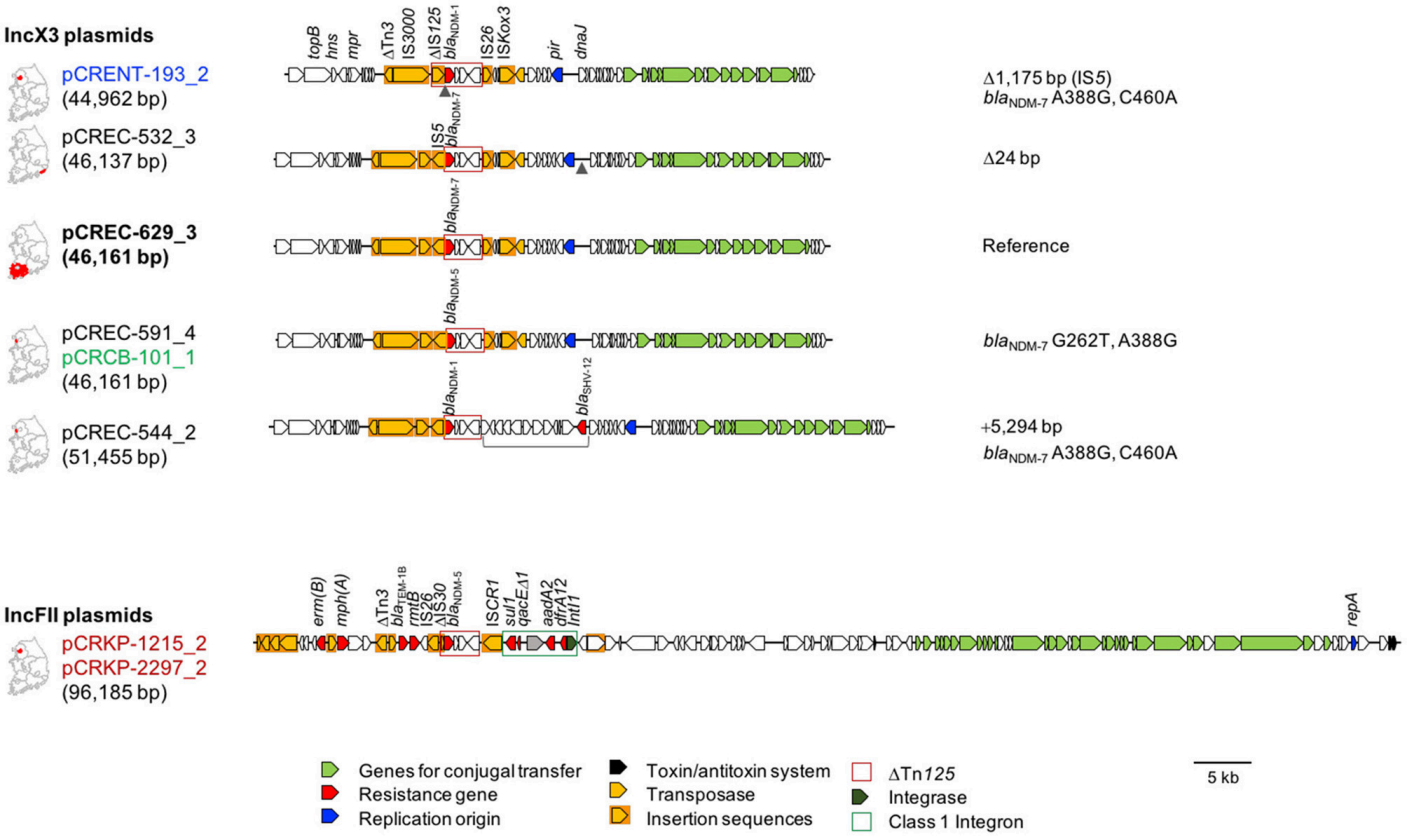
Schematic representation of the *bla*_NDM_-harboring IncX3 and IncFII plasmids. Name of the plasmids and the size in parenthesis are denoted for the plasmids, and the district of isolation are indicated on the map of Korean peninsula in front of the plasmid name. Name of the plasmids from *E. coli* are indicated in black, from *Enterobacter* spp. in blue, and from *K. pneumoniae* in red. Open arrows indicate coding sequences for conjugal transfer (light green), drug resistance (red), replication origin (blue), toxin/antitoxin system (black), transposase (yellow), and integrase (green). Insertion sequences are indicated in yellow boxes. The Tn*125* is marked with a red-bordered open box, and the class 1 integron is indicated with a green-bordered open box. Genetic variants of each plasmid referred by pCREC-629_3 are indicated on the right side of the schematic representation of the plasmid for locating the point of substitution: arrowheads, deletion; square bracket, insertion. Together with the nt substitutions, details of insertion are indicated with +, and that of deletion with Δ in the end of the schematic presentation of the plasmid.

As the IncX3 plasmids were the most common among NDM producers, regardless of the NDM subtype or bacterial host, we completely sequenced a panel of six NDM producers that possessed IncX3 plasmids carrying any of three NDM subtypes (Figure 1, Table 4). Two OXA-181 and NDM-5 co-producers in which the *bla*_NDM−5_ gene was carried by the IncFII plasmid were added to the panel for comparative analysis. The eight isolates exhibited MDR or XDR resistance phenotypes (Figure 1, Table 4). The *Enterobacter* sp. CRENT-193 was an isolate in 2013, while *K. pneumoniae* CRKP-1215 was in 2014, and the remaining six were in 2015 (Table 4). Three isolates (*Enterobacter* sp. CRENT-193, *K. pneumoniae* CRKP-1215, and CRKP-2297) were from Seoul, among which both *K. pneumoniae* isolates co-producing OXA-181 and NDM-5 were from one hospital with one-year interval. Two *E. coli* isolates (CREC-544 and CREC-591) and *C. freundii* CRCB-101 were from Incheon, a suburb of Seoul, among which *E. coli* CREC-591 and *C. freundii* CRCB-101 were from a single hospital outbreak. The remaining *E. coli* CREC-532 and CREC-629 isolates were recovered in the southwestern and southeastern peninsula, respectively.

**Table 4:** Antimicrobial determinants in the chromosomes and plasmids of entirely sequenced NDM producers^a^.

**Isolate [MLST]^b^**	**Isolationyear/** **region-hospital/** **specimen**	**Resistance phenotype^c^MICs (mg/L) of imipenem/** **meropenem**	**Chromosome (GenBank acc.)**	**Plasmids (GenBank acc.)**									
				**IncFII**		**IncFII-FIA-FII**	**IncFIB-FIC**	**IncI1**	**IncA/C2**	**IncHI2A-HI2**	**IncX3**	**IncY**	**Non-typable**
*Enterobacter* sp. CRENT-193	2013/ Seoul-A/ Wound	ATM-CTX-CAZ-FOX-GM-AN-CIP 64/64	4,852,690 bp (CP024812) *fosA* *bla*_ACT−16_ *aadA1* *sul1* *dfrA1*	-	-	-	-	-	-	298,989 bp (CP024813) *tet(A)* *dfrA14* *qnrB1* *aac(6')-Ib_*cr*_* *bla*_OXA−1_ *catB3* *aac(3)-IIa* *bla*_CTX−M−15_ *bla*_TEM−1B_ *strAB* *sul2* *aadA1* *catA1*	**44,962 bp** (CP042814) ***bla***_**NDM−1**_	-	-
*K. pneumoniae* CRKP-1215 [ST147]	2014/ Seoul-B/ Bronchial washing	ATM-CTX-CAZ-FOX-GM-AN-CIP 128/>128	5,420,542 bp (CP024838) *bla*_OXA−181_ *fosA* *bla*_OXA−181_ *bla*_CTX−M−15_ *oqxAB* *rmtf* *aac(6')Ib-cr* *aacA4* *arr-2* *bla*_SHV−11_	130,922 bp (CP024839) *arr-2* *qnrB66* *aadA2* *dfrA12* *catA2* *rmtf* *aacA4*	**96,185 bp** (CP024840) *dfrA12* *aadA2* *sul1* ***bla***_**NDM−5**_ *rmtB* *bla*_TEM−1B_ *mph(A)* *erm(B)*	-	-	-	-	-	-	-	72,689 bp (CP024841) NRD
*K. pneumoniae* CRKP-2297 [ST147]	2014/ Seoul-B/ Bronchial washing	ATM-CTX-CAZ-FOX–AN-CIP-TGC 64/>128	5,429,958 bp (CP024834) *bla*_OXA−181_ *fosA* *bla*_OXA−181_ *bla*_CTX−M−15_ *oqxAB* *rmtf* *aacA4* *arr-2* *bla*_SHV−11_	112,150 bp (CP024835) *arr-2* *qnrB66*	**96,185 bp** (CP024836) *dfrA12* *aadA2* *sul1* ***bla***_**NDM−5**_ *rmtB* *bla*_TEM−1B_ *mph(A)* *erm(B)*	-	-	-	-	-	-	-	69,628 bp (CP024837) NRD
*E. coli* CREC-544 [ST457]	2015/Incheon-C/Rectal swab	ATM-CTX-CAZ-FOX-CIP 16/16	4,903,571 bp (CP024826) *bla*_CTX−M−55_	-	-	-	122,937 bp (CP024827) *aph(3')-Ia* *catA2* *dfrA14* *sul2* *strAB* *bla*_TEM−1B_ *bla*_CTX−M−55_	-	-	-	**51,455 bp** (CP024828) ***bla***_**NDM−1**_ *bla*_SHV−12_	-	32,182 bp (CP024829) NRD
*E. coli* CREC-591 [ST101]	2015/ Incheon-D/ Peritoneal fluid	ATM-CTX-CAZ-FOX-CIP 32/64	5,125,266 bp (CP024821) *catA1* *dfrA14* *mph(A)* *aph(3')-Ia* *tet(A)*	64,564 bp (CP024824) *fosA* *bla*_TEM−1B_ *bla*_CTX−M−55_	-	-	-	118,156 bp (CP024822) *mph(A)* *fosA* *bla*_CTX−M−123_	62,815 bp (CP024823) *sul2* *strAB* *tet(A)* *floR*	-	**46,161 bp** (CP024825) ***bla***_**NDM−5**_	-	
*C. freundii* CRCB-101	2015/ Incheon-D/ Open pus	ATM-CTX-CAZ-FOX 16/16	5,311,319 bp (CP024819) *bla*_CFE−1_ *qnrB28*	-	-	-	-	-	-	-	**46,161 bp** (CP024820) ***bla***_**NDM−5**_	-	-
*E. coli* CREC-532 [ST167]	2015/ Busan-E/ Urine	ATM-CTX-CAZ-FOX-GM-CIP >128/>128	4,837,992 bp (CP024830) *bla*_CTX−M−55_ *bla*_CTX−M−14_ *tet(B)*	-	-	216,181 bp (CP024831) *aac(3)-IId* *dfrA17* *aadA5* *sul1* *mph(A)* *erm(B)* *tet(B)* *aac(3)-IId* *bla*_TEM−1B_	-	-	-	-	**46,137 bp** (CP024833) ***bla***_**NDM−7**_	96,987 bp (CP024832) NRD	-
*E. coli* CREC-629 [ST167]	2015/ Jeonnam-F	ATM-CTX-CAZ-FOX-GM-CIP 16/32	4,938,993 bp (CP024815) *bla*_CTX−M−55_ *bla*_CTX−M−14_ *tet(B)*	-	-	176,274 bp (CP024816) *bla*_TEM−1B_ *aac(3)-IId* *tet(B)* *erm(B)* *mph(A)* *sul1* *aadA5* *dfrA17*	-	-	-	-	**46,161 bp** (CP024818) ***bla***_**NDM−7**_	96,990 bp (CP024817) NRD	-

a *The sizes of the chromosomes and plasmids are indicated, together with resistance determinants identified. NRD, No resistance determinants were identified in the plasmid*.

b *STs of E. coli and K. pneumoniae are indicated*.

c *ATM, aztreonam; CTX, cefotaxime; CAZ, ceftazidime, FOX, cefoxitine; GM, gentamicin; AN, amikacin; CIP, ciprofloxacin; and TGC, tigecycline*.

*The blaNDM genes were indicated in bold together with the size of plasmid possessing the gene*.

The IncX3 plasmids harboring *bla*_NDM−5_ or *bla*_NDM−7_ had consistent sizes of 46,137 and 46,161 bp, respectively, whereas the two plasmids harboring the *bla*_NDM−1_ gene were 44,962- and 51,455-bp long. The six IncX3 plasmids had conserved backbones including elements of conjugal transfer, the replication origin, and other accessory genes. The differing lengths of the plasmids were due to the excision of a 1,175-bp ΔIS*5* copy upstream of the truncated Tn*125* in pCRENT-193_3 and the insertion of a 5,294-bp fragment including *bla*_SHV−12_ in pCREC-544_2 (Figure 1). Of note, the plasmids pCREC-591_4 and pCRCB-101_1, which were identified from different pathogenic species (*E. coli* ST101 and *C. freundii*) in a single hospital outbreak, were identical, clarifying the inter-species transfer of the plasmid. The six isolates possessed various resistance determinants in their chromosomes. Interestingly, CTX-M-type ESBLs and plasmid-mediated AmpCs, which are known to be more common in plasmids than in the chromosome, were frequently encoded in the chromosome (Table 4). Further, except for *C. freundii* CRCB-101, all isolates co-carried additional plasmids with multiple genes for drug resistance (Table 4).

Both IncFII plasmids (pCRKP-1215_2 and pCRKP-2297_2) from *K. pneumoniae* ST147 isolates recovered at a hospital were identical, even they were identified one year apart. The co-produced OXA-181 was encoded in the chromosome by two copies of *bla*_OXA−181_ and the chromosomes of the two isolates were very similar to each other, sharing 99% nt identity on 100% coverage. Taken together, these results clearly demonstrate the persistence of the clone in the clinical setting. The IncFII plasmid itself conferred MDR, due to the *aadA3, dfrA23*, and *sul1* genes associated with the class 1 integron, the *bla*_TEM−1B_ accompanied by Tn*3*, and the *erm(B), mph(A)*, and *rmtB* genes (Figure 1 and Table 4). A set of three plasmids (two different IncFII and one non-typeable) was carried together by the isolates.

## Discussion

The epidemiology of NDM-mediated carbapenem resistance is complex because the dissemination involves multiple factors, such as the clonal spread of resistant strains and the inter-strain and inter-species horizontal transfer of the resistance determinants. The global NDM producers include diverse species and genera within Gram-negative bacteria and a wide range of clones within individual species, as indicated by ST. The NDM-encoding plasmids and the mobile elements of the *bla*_NDM_ gene are also complicated in South Korea depend on the bacterial species and the strains. The mobility of the plasmid is critical for the dissemination of *bla*_NDM_. NDM-producing clones possessing a transferable plasmid with *bla*_NDM_ have been demonstrated to spread *bla*_NDM_ efficiently, while a non-conjugative plasmid is reliable for clonal spread (Kumarasamy et al., 2010).

Until 2012, the occurrence of NDM producers in South Korea was regionally restricted and the constituents were simple. Beginning in 2013, the NDM producers became diverse at the species level and in 2014, a range of varied clones began to be identified within individual species. Finally, in 2015, two additional NDM variants were recognized within even more diverse bacterial hosts. Thus, the population of NDM producers in South Korea achieved the epidemiological complexity and diversity as worldwide NDM population. The IncX3 plasmid carrying the *bla*_NDM_ gene, which is either transferable or non-mobile, was likely responsible for the dissemination of NDM producers over the peninsula.

The six circularized IncX3 plasmids had a relatively simple structure composed of conjugal transfer elements, accessory genes, and a single resistance determinant (*bla*_NDM_). Only one IncX3 plasmid possessed one *bla*_SHV−12_ gene in addition to the *bla*_NDM_ gene. The IncX3 plasmids identified in this study were identical to those from the Middle East, which is a recently added reservoir of NDM producers (Zowawi et al., 2013). This simple plasmid, in terms of resistance gene carriages, has a broad host range, which greatly enhances its inter-genera and inter-species dissemination. Actually, in our collection of isolates, more than three-quarters of the *bla*_NDM_-possessing plasmids were IncX3 type and were carried by varied clones of various species of bacteria.

All NDM producers were resistant to more than three classes of antimicrobial drugs. Further, the NDM producers, especially those possessing the IncX3-type *bla*_NDM_ plasmid, were vested in MDR, either due to supplemental plasmid(s) containing several genes for antimicrobial resistance, as we observed in this study, or due to the hybridization of plasmids with additional resistance genes (Sun et al., 2016). The IncA/C- and IncFII-type plasmids were kept within *E. coli* and *K. pneumoniae* bacterial hosts, though they are known to have broad host ranges; thus, their spreads were limited both temporally and regionally.

The NDM-5 and NDM-7 producers, even with relatively small numbers of isolates, had higher carbapenem MICs than the NDM-1 producers (Table 1). These subtypes share a substitution of Methionine to Leucine at position 154, identical to that in NDM-4 (Nordmann et al., 2012), and have greater MICs than NDM-1, not only for carbapenems, but also for 3rd- and 4th-generation cephalosporins such as cefotaxime, ceftazidime, and cefpirome (Hornsey et al., 2011). Additionally, as shown by both entirely sequenced co-producers, co-production of OXA-181 with NDM-5, could contribute to elevate the carbapenem MICs. Notably, the *bla*_NDM−5_ gene, which was observed in both IncX3 and IncFII plasmids, was initially identified in an IncF-type plasmid possessed by *E. coli* ST648 (Hornsey et al., 2011). And the *bla*_NDM−7_ gene, which was identified only in the IncX3 plasmid, was first identified in the IncX3 plasmid (Göttig et al., 2013).

It is noteworthy that a quite systematized alliance with other antibiotic resistance determinants has been observed in almost all NDM producers. The additional antimicrobial resistance genes for plasmid-mediated AmpC cephalosporinases, CTX-M-type ESBLs, and 16S RNA methyltransferases have enhanced the MDR of NDM producers. This strategy has facilitated the persistence of pathogens that have encountered combination antimicrobial therapies in clinical settings.

Clonal dissemination played a major role in the dissemination of NDM producers in South Korea and the IncX3 plasmid possessing the *bla*_NDM_ gene collaborated for the gene spread, at least among the entirely sequenced strains. NDM producers pose a major healthcare risk since the isolates are usually MDR, and the plasmids are frequently transferable to a wide range of Gram-negative bacteria. This time-course observation of the genomic epidemiology of NDM has warned us of the great public health threat of NDM producers, raising the importance of national policy to combat drug resistance.

## Ethics statement

The research, which has no involvement of human subject but the clinical isolates, does meet the exempt category without approval from Ethics Committee on Human Research of the Health Ministry in South Korea and the study design has not been reviewed by the committee.

## Author contributions

KL and SJ conceived the project. E-JY and SJ wrote the manuscript. E-JY, DaK, and JY performed the experiment. E-JY, SJ, and KL analyzed and interpreted the data. JY, DoK, HL, and KL maintained the collections.

### Conflict of interest statement

The authors declare that the research was conducted in the absence of any commercial or financial relationships that could be construed as a potential conflict of interest.
