# Supplementary material for: New Delhi Metallo-Beta-Lactamase-Producing Enterobacteriaceae in South Korea Between 2010 and 2015
Source: Front Microbiol. 2018 Mar 29;9:571. doi: 10.3389/fmicb.2018.00571 (PMC5884925; doi:10.3389/fmicb.2018.00571)
Supplement: Supplementary file 1 [file Image1.PDF]

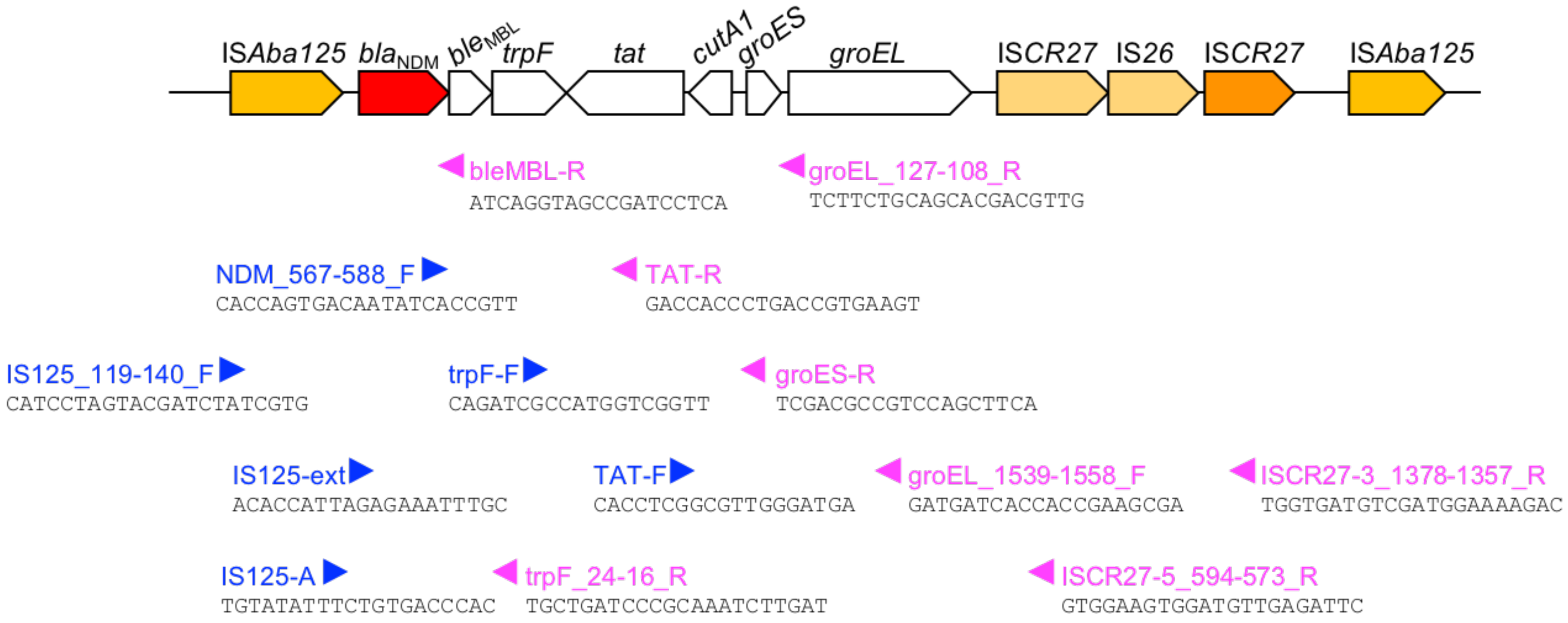

Supplemental Figure. Primers used for PCR mapping to explore the vicinity of the *bla*$_{NDM}$ gene. Arrows represents open reading frames and the name of each gene is indicated above the arrow. Blue arrowhead indicates the location of forward primers and magenta arrowhead indicates that of reverse primers. Primer sequences are represented below the name of each primer.
